# Periscope

**Published:** 1888-06

**Authors:** 


### PERISCOPE.

### MEDICINE.

**The Bacillus of Epidemic Dysentery.**—MM. Chanteuresse and Widal have obtained the following results from a study of five cases of dysentery contracted in hot countries. First of all, they performed an autopsy at Algiers on a soldier who had just died of an acute exacerbation of a dysentery contracted in Tonquin. In the fæcal matters during life, in the walls of the large intestine, in the mesenteric glands, and in the spleen after death, they found a bacillus which is not present in healthy dejections, but which also constantly appeared in the stools of four other dysenteric patients. The lesions in the case of the soldier above mentioned were characteristic of dysentery, and the bacillus was found in masses on the surface of the mucous membrane, in the intestinal walls, between the tubular glands, and lying in these at their terminations. From the mucous membrane, spleen and mesenteric glands they succeeded in obtaining pure cultures of a micro-organism which grew readily on gelatine at the ordinary temperature. The microbe formed a rod rounded at the extremity. Its transverse diameter increases in successive cultures; it stains badly with aniline dyes, and does not liquefy the gelatine, forming a whitish pellicle on the surface which does not touch the sides of the glass. It grew with great rapidity in sterilised water from the Seine. Isolated colonies in gelatine plate cultures at one period of their development assume a characteristic appearance useful in diagnosis. When they are hardly visible to the naked eye, under a lens they appear as a clean spot. A little later they have a yellowish tinge, and appear to be made up of two concentric circles. Later still the yellow colour is lost, and they become whitish and granular. The diameter of these colonies never exceeds that of a lentil. The authors never detected the formation of spores.

The bacilli were given to rabbits by the mouth, by inoculation of the intestine, and by intra-peritoneal injection. When injected by the mouth there were no effects for the first few days, but on killing the animal on the eighth day the stomach was found to be studded with small ulcers about the size of a lentil. The ascending colon was distended, and contained liquid fæcal matter, with abundance of the injected microbes; its walls were thickened and contained numerous ecchymoses, and the lymphoid follicles were atrophied. When the acidity of the gastric juice was neutralised by administering carbonate of soda before giving the microbe, the gastric lesions were much more pronounced, consisting of large irregular ulcerated surfaces, covered with a pultaceous false membrane resting upon an indurated whitish base. Intra-peritoneal inoculation caused death in from two to three days with pericarditis, peritonitis and pleurisy, with formation of much fibrin. The bacilli were found in the blood and in the fibrinous exudations.

Intra-intestinal inoculation after laparotomy gave the most decisive results. In animals killed at the end of eight days the large intestine was filled with liquid fæcal matter containing the bacillus. The mucous membrane was swollen, ecchymosed and ulcerated. Under the microscope the lesions appeared to be disseminated in patches isolated from each other, and situated most frequently in the lymphoid follicles. The central part of the lesion consisted of a coagulation necrosis, and in the capillaries around were swarms of bacilli similar to those injected: the bacilli also penetrated the mucous membrane along the connective tissue between the tubular glands. The liver contained two or three patches in which the gland-tissue had become yellow. Pure cultures obtained from the intestinal lesions reproduced the bacillus inoculated eight days before. The authors conclude that the presence of the bacillus above described in the intestinal lesions, glands, and spleen of a man dying from an acute attack ot dysentery, its absence in healthy evacuations, and the lesions it produces in the intestine and viscera of rabbits justify the assumption that it is the specific bacillus of dysentery.—*Le Progrès médical*, April 21st, 1888.

**Comparison between the use of Antipyrin and of Salicylate of Soda in painful affections, and especially in those of Rheumatic origin, by Dr. Chas. Bayoux.**—In acute rheumatism salicylate of soda gives excellent results, provided that the kidneys are sound and the nervous system not too excitable: on the other hand, when the patient has albuminuria, or is very nervous, especially if a woman, its use is often attended with inconvenience. In these cases antipyrin may be administered with advantage. In cases of chronic or of muscular rheumatism, and in various neuralgias, the salicylates are ineffectual, while antipyrin gives excellent results. Generally speaking, antipyrin is better tolerated, is less toxic than salicylate of soda, and has no injurious action on the renal epithelium. The nausea and vertigo that it occasionally produces speedily disappear, whilst the erythema it sometimes gives rise to is of slight intensity and short duration. Accidents can be reduced to a minimum by employing only chemically pure antipyrin, and varying the mode of administration (by giving it in pill, mixture, by hypodermic injection, or by enemata).—*Le Progrès médical*, April 21st, 1888.

**Treatment of Hæmoptysis in Phthisis by Iodoform.**—MM. Chauvin and Jovissenne, of Liège, give fourteen cases in which the administration of iodoform for hæmoptysis was attended with immediate and marked success. In most of the cases the hæmoptysis did not occur in quantity, but in the form of expectoration, more or less abundant, constantly streaked with blood. From their observations they draw the following conclusions:—

(1) Iodoform is a rapid and certain hæmostatic in cases both of severe loss of blood and in sanguineous expectoration.

(2) Relapses are very rare; no case of relapse or of death from hæmoptysis occurring after ten months, although the patients were very poor, and lived in a populous quarter amidst bad hygienic surroundings.

(3) The dose required is small. They gave the drug in pills each containing $\frac{3}{4}$ grain. Three pills in the twenty-four hours were generally sufficient: more than nine were very rarely required. At first they combined the iodoform with tannin, but soon found that the tannin was unnecessary.

(4) Iodoform succeeded where ergotin completely failed, and had the further advantages of not disturbing the digestion, and permitting the patients to continue their occupations.

The manner in which the hæmostatic action of iodoform is produced they hope to discuss in a future paper, but at present have not exactly determined.—*Le Progrès médical*, May 19th, 1888.

**Indications and Contra-Indications for the use of the Contrexeville Waters.**—Dr. Bayard has made the following observations on the action of the Contrexéville waters during twenty years' practice in that place:

The waters possess two properties: the first is called "expulsive" from its action on plain muscular tissue, and especially that of the biliary and urinary passages; the second, or "alterative," affects the general composition of the blood like other alkalies.

To the first action is to be attributed the expulsion of stones from the bladder that often occurs while the patient is undergoing a course of the waters, and the success attained in incontinence of urine in children, and in vesical catarrh with enfeebled contractile power in the aged.

Its alterative action explains its good effects in gout and diabetes, where it acts by facilitating the excretion of uric acid.

Indications:

(1) Uric acid, phosphatic or oxalic gravel—above all, if there are attacks of renal colic: renal hæmaturia or alkalinity of urine is not a contra-indication.

(2) Vesical catarrh: many of the most remarkable cures brought about by the waters are in cases of this description.

(3) Although successful in acute gout, the waters are more especially valuable in chronic cases.

(4) According to the author's experience, the sugar disappears from the urine in 89 per cent. of cases of diabetes.

(5) Hepatic colic: the best results are obtained in women whom frequent attacks of colic have rendered weak and anæmic.

Contra-indications:

(1) The presence of a stone in the bladder too large to be passed by the urethra.

(2) Complete paralysis of the bladder, with absolute loss of all contractility.

(3) Organic disease of the heart demands the greatest care in the use of the Contrexéville waters.

Finally, the author recommends that the waters should always be taken fasting, since, if taken at meal times, they may fail to produce good effects.—*Gazette des hôpitaux*, March 10th and 12th, 1888.

[The Contrexéville waters approach closely in composition the sulphated lime waters of Bath and Clifton: the great reputation that the latter possessed for cases of diabetes, during the 18th and early part of the 19th centuries, will be remembered.] J. MICHELL CLARKE, M.B.

### LARYNGOLOGY.

**Varix of the Tongue.**—Dr. Manon considers that the condition depends on a gouty or rheumatic diathesis as its predisposing cause: it is rare in infants, but fairly common in barristers, singers, street hawkers, and those addicted to alcohol. The seat of the disease is behind the circumvallate papillæ.

The symptoms are: a little hindrance to the movements of the organ and to clear speech, dryness of the throat, production of sputum difficult to expel, and which is sometimes pink.

The treatment consists in removal by galvano-cautery or curved scissors.—*Revue mensuelle de Laryngologie*, April, 1888.

[I have just seen a case of early phthisis laryngea in a patient addicted to alcoholic excess, in which, along with much overfilled and varicose veins, there were considerable outgrowths, like small angiomata, on the back of the tongue and stretching down to the epiglottis. The best way to see these is to hold the laryngoscopic mirror higher in the throat than one would do in an ordinary inspection of the larynx.]

**Tuberculosis of the Tonsils.**—Dr. Lublinski finds this to be extremely rare; but since Strassmann, Cornil and Ranvier and Ziegler have found small tubercle follicles in the tonsils of those who have died of phthisis pulmonalis, but who during life gave no sign of any ailment of the tonsils, it is probably not so rare a disease in the hands of pathologists as in those of physicians.

In Lublinski's two cases, the lungs and larynx were invaded, and then ulceration of tonsils took place without the neighbouring structures being affected. The ulcers are like those caused by tubercle attacking a mucous surface; and numerous bacilli were present, and these Lublinski considers of great importance, as otherwise the appearances might be confounded with those of syphilis. The treatment was palliative by means of cocaine, and also iodol was used. The galvano-cautery appeared to relieve one case. The author thinks that the development of new connective tissue conceals the seat of tubercle and so hides it from the clinical observer. It is probable that the infection of the tonsils occurs through the expectoration.—*Revue mensuelle de Laryngologie*, April, 1888.

**The Action of Caustics on the Nasal Mucous Membrane.**—Dr. Francke H. Bosworth explains the action of these substances in hypertrophic rhinitis by referring us to the pathology of the disease, which he believes to be a dilatation of venous sinuses, increased blood-supply, hypernutrition and therefore increase of the intervenous connective tissue.

He states that normally sixteen ounces of clear serum are daily poured into the nasal cavities from the venous sinuses; if the walls of the vessels are thickened this amount is lessened, and a thick, annoying discharge takes the place of the natural fluid.

Our object in using remedies is to constrict vessels and thus counteract hypertrophy. Caustics cause necrosis of superficial epithelium, and to a very slight degree of the submucosa; they do not affect the deep cavernous layer, but the superficial slough formed compresses the blood-vessels. Now, cocaine causes depletion of blood-vsssels by diminishing their calibre; if, then, we get this effect first and then use a caustic, we shall do more to maintain the vessels in a state of contraction until they can regain their normal tonicity, than if we omit the cocaine. Bosworth considers chromic acid the best caustic to use, because, although temporarily the pain caused by it is just as great as that caused by a mineral acid, it more quickly and surely subsides, does not produce cicatrices, and with care, its action can be easily controlled.—*The Journal of Laryngology*, April, 1888.

**Cocaine as a hæmostatic in Epistaxis and in Nasal Surgery.**—Rualt uses tampons soaked in 20 to 30 per cent. solutions of cocaine, which, in obstinate cases of epistaxis, he allows to remain in the nose two or three hours. In bleeding produced by surgical interference, five or ten minutes are usually enough.—*Revue mensuelle de Laryngologie*, February, 1888.

**The action of Cocaine on Laryngeal Infiltrations, an aid to Diagnosis.**—Baumgarten considers that if an infiltration becomes smaller and paler after cocaine has been applied to it, it is catarrhal; if there is no change, it is more serious.—*Wiener med. Wochenschrift*, No. 44, 1887.

**Pachydermia verrucosa of Larynx.**—Professor Zenker reports a case of the above rare condition: both vocal cords were much thickened, with milk-white colour of the surface which was smooth; on vertical section through the cord, he found long, closely-packed papillæ, which in some cases were branching, and covered by a thick layer of epithelial cells, which presented an even surface; under the papillæ there was an inflammatory small cell-infiltration, the cells of which were quite unlike an epithelial collection.

Zenker considers such a growth as quite innocent.—*Münch. med. Wochenschrift*, No. 32, 1887.

BARCLAY J. BARON, M.B.

